# COPD and bronchiectasis; more than mere enemies

**DOI:** 10.36416/1806-3756/e20250086

**Published:** 2025-05-21

**Authors:** Grace Oscullo, Amina Bekki, Miguel Ángel Martinez-García

**Affiliations:** 1 Servicio de Neumología e Instituto de Investigación La Fe - IISLAFE - Hospital Universitario y Politécnico La Fe, Valencia, España.; 2 Centro de Investigación Biomédica en Red de Enfermedades Respiratorias, Instituto de Salud Carlos III. Madrid, España.

COPD, asthma, and bronchiectasis are currently the three most common chronic inflammatory diseases of the airways.^1^ ^-^ ^3^ However, it has not always been this way. COPD and asthma have been known for decades, but bronchiectasis began to be clearly visible in the 1980s when Naidich et al.^4^ defined it based on CT images and later on its high-resolution forms. Until then, no one suspected that there could be any relationship between bronchiectasis, COPD, and asthma. The first paper that found a relationship between bronchiectasis and COPD dates back to 2003, when a group in London observed that 50% of patients with severe COPD presented with radiological bronchiectasis, longer exacerbations, and greater bronchial inflammation, as well as a greater quantity of bronchial pathogens.^5^ We are therefore faced with a story that began about 20 years ago. This development attracted a lot of attention from the scientific community, since the first questions that appeared to require an answer were, obviously, as follows. Is “this” bronchiectasis caused by COPD itself or by other etiologies? And, most importantly, does the presence of bronchiectasis alter the prognosis and treatment of patients with COPD?

A search in PubMed for studies that have addressed this issue about the relationship between bronchiectasis and COPD shows that the research on this relationship has skyrocketed significantly in the past 8 years. All of this has led, nowadays, to the main international regulations on COPD^6^ ^,^ ^7^ and bronchiectasis^8^ ^,^ ^9^ considering this combination as a special phenotype, given the high prevalence of bronchiectasis in COPD and the negative prognosis it produces. This conclusion is based on dozens of studies and two meta-analyses,^10^ ^,^ ^11^ which show that the prevalence of bronchiectasis in COPD is between 6% and 57% and that it increases with greater severity of COPD in terms of airflow obstruction, just as the presence of pathogenic microorganisms, the clearest cause of the onset of bronchiectasis, is also greater in severe forms of COPD.

Of all these studies, we might highlight four. The first, published in 2013 and one of the oldest, a multicenter study, was the first to demonstrate that bronchiectasis not only caused greater severity of COPD, but also doubled the probability of death by 2.5, after adjustments.^12^

Two subsequent meta-analyses published in 2015 and 2016, respectively,^10^ ^,^ ^11^ delved deeper into these conclusions. Ni et al.^10^ observed, in a group of 881 patients with COPD, that the mean prevalence of bronchiectasis was 54% (95% CI: 25-69%), similarly to previous studies; this was higher in those with greater severity of COPD, especially in men with a longer history of smoking and a greater quantity of and purulence in sputum, greater purulence in expectoration, worse lung function, greater systemic inflammation, and greater chronic infection by pathogenic microorganisms, especially *Pseudomonas aeruginosa*. For their part, Du et al.,^11^ in addition to confirming these results, went further and confirmed the previously observed results of the doubling of the adjusted mortality in these patients with COPD. Therefore, the high prevalence of bronchiectasis in COPD, the impact on the prognosis, and the different treatments that bronchiectasis require for these patients-it is necessary to remember that the treatment of bronchiectasis, unlike that of COPD, is based on antibiotics, and there is a relative contraindication of inhaled corticosteroids-meant that this type of patient could be identified as having the COPD-bronchiectasis phenotype, as it appears in the guidelines for both diseases.^8^ ^,^ ^13^ Moreover, this phenotype was supported by a particular endotype, since these patients presented with a greater amount of mucins, greater neutrophilic inflammation, and gram-negative infection in the airways.^14^ ^,^ ^15^

**Figure 1:**
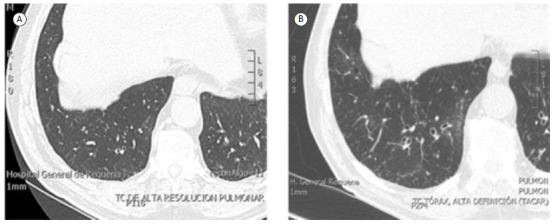
CT scans of a patient without bronchiectasis (A) and showing the development of bronchiectasis seven years later (B). With permission of the Journal of Clinical Infectious Disease.

However, the last question remained unresolved. Is COPD *per se* the cause of bronchiectasis? Or, in other words, is bronchiectasis a normal part of the natural history of COPD? Although the general recommendation is that the appearance of bronchiectasis in patients with COPD should not always be sought for a cause other than COPD, it was observed, in a well-designed study,^16^ that 19.5% of patients with COPD without bronchiectasis had radiological bronchiectasis and compatible symptoms after 8.5 years of follow-up, and “this” bronchiectasis could not be attributed to other etiologies (Figure 1). For the first time, a causal relationship was established between the two diseases.^16^

Finally, with regard to treatment, the guidelines^8^ ^,^ ^13^ recommend that both diseases should be treated, but there is a double dilemma that has yet to be resolved. On the one hand, treatment with inhaled corticosteroids in patients with COPD and bronchiectasis can increase the risk of pneumonia; so, if they have to be used, minimal doses are recommended or should be replaced with macrolides when possible. On the other hand, there is a long-term treatment with inhaled antibiotics for COPD patients with chronic bronchial infection without bronchiectasis to prevent the onset of the latter, although this remains under study.

We can also think the other way around. What percentage of patients with a major diagnosis of bronchiectasis has COPD? According to an analysis of several bronchiectasis registries around the world, this is between 3.4% and 20%, with little information on how COPD can affect the prognosis of bronchiectasis, although a study by a Spanish bronchiectasis group shows that the diagnosis of COPD in these patients has increased significantly, from 7% to 12% overtime.^9^

Therefore, it is currently recommended that in COPD patients with multiple exacerbations, repeated isolation of pathogenic microorganisms in respiratory samples, especially *P. aeruginosa*, a too rapid decline in lung function, poor clinical evolution, or severe COPD, a CT scan should be performed in search of bronchiectasis^7^ ^,^ ^8^ ^,^ ^13^ regardless of whether this is due to COPD itself or not; both the prognosis and the evolution/treatment of the patients might change, and bronchiectasis can therefore become another treatable trait in these patients.
